# Predictors of high-cost hospitalization in the treatment of acute coronary syndrome in Asia: findings from EPICOR Asia

**DOI:** 10.1186/s12872-018-0859-4

**Published:** 2018-07-04

**Authors:** Stephen Jan, Stephen W-L. Lee, Jitendra P. S. Sawhney, Tiong K. Ong, Chee Tang Chin, Hyo-Soo Kim, Rungroj Krittayaphong, Vo T. Nhan, Stuart J. Pocock, Ana M. Vega, Nobuya Hayashi, Yong Huo

**Affiliations:** 1 The George Institute for Global Health, Sydney Medical School, University of Sydney, King George V Building, 83–117 Missenden Rd, Camperdown, NSW 2050 Australia; 2 Queen Mary Hospital, Hong Kong, SAR China; 3 Sir Ganga Ram Hospital, New Delhi, India; 4 Sarawak General Hospital, Kuching, Malaysia; 5 National Heart Centre Singapore, Singapore, Singapore; 6 Seoul National University Hospital, Seoul, South Korea; 7 Siriraj Hospital, Bangkok, Thailand; 8 Cho Ray Hospital, Ho Chi Minh City, Vietnam; 9 London School of Hygiene and Tropical Medicine, London, UK; 10 Observational Research Centre, Global Medical Affairs, AstraZeneca, Madrid, Spain; 11 AstraZeneca, Osaka, Japan; 12 Peking University First Hospital, Beijing, China

**Keywords:** Acute coronary syndrome, Asia, Health insurance, Costs, Hospitalization

## Abstract

**Background:**

The EPICOR Asia (long-tErm follow-uP of antithrombotic management patterns In acute CORonary syndrome patients in Asia) study (NCT01361386) was an observational study of patients hospitalized for acute coronary syndromes (ACS) enrolled in 218 hospitals in eight countries/regions in Asia. This study examined costs, length of stay and the predictors of high costs during an ACS hospitalization.

**Methods and results:**

Data for patients hospitalized for an ACS (*n* = 12,922) were collected on demographics, medical history, event characteristics, socioeconomic and insurance status at discharge. Patients were followed up at 6 weeks’ post-hospitalization for an ACS event to assess associated treatment costs from a health sector perspective. Primary outcome was the incurring of costs in the highest quintile by country and index event diagnosis, and identification of associated predictors. Cost data were available for 10,819 patients. Mean length of stay was 10.1 days. The highest-cost countries were China, Singapore, and South Korea. Significant predictors of high-cost care were age, male sex, income, country, prior disease history, hospitalization in 3 months before index event, no dependency before index event, having an invasive procedure, hospital type and length of stay.

**Conclusions:**

Substantial variability exists in healthcare costs for hospitalized ACS patients across Asia. Of concern is the observation that the highest costs were reported in China, given the rapidly increasing numbers of procedures in recent years.

**Trial registration:**

NCT01361386 *.*

**Electronic supplementary material:**

The online version of this article (10.1186/s12872-018-0859-4) contains supplementary material, which is available to authorized users.

## Background

Ischemic heart disease is associated with a substantial healthcare burden worldwide, in terms of both deaths and disability-adjusted life years [1], while the costs of treating acute coronary syndromes (ACS) also represent a major burden for healthcare systems globally [2–6]. With the increasing prevalence of lifestyle-related chronic-disease risk factors, increasing healthcare costs, technological innovation, and growing consumer and patient expectations regarding access to twenty-first century healthcare, it is expected this burden will likely continue to increase [5]. This will have implications for the future financial sustainability of healthcare systems globally. These pressures are particularly pronounced in low- and middle-income countries in Asia where large-scale policies are underway or in planning; notably, in India and China to achieve universal coverage through the expansion of financial coverage for treatments to previously under-served populations. Such reforms, while critical in promoting social protection and equity of access to healthcare, come with significant resource requirements and will inevitably magnify future health sector financing challenges.

In addressing issues of cost and financial sustainability, understanding of the factors that drive variation in resource use is important, particularly those factors that contribute to higher treatment costs. Notably, only very few studies have examined the factors associated with variation in treatment costs for ACS. Evidence from the US, comparing inpatient resource use for ACS in patients who died in hospital with that for a surviving ACS cohort, indicated that inpatient mortality for ACS is associated with a 47% greater duration of hospital stay along with an incremental cost of around US$43,000 [7]. Similarly, in a study conducted in Italy, which followed up patients for 12 months’ post-hospitalization for ACS, patients who died of a cardiovascular event had an average cost of around €16,000 compared with an average cost of around €11,000 for the entire ACS cohort [8]. In China, a small study in a single hospital in Shandong found that increased age was associated with increased treatment costs and poorer clinical outcomes [9]. In a study in the US of over 12,000 patients with ACS, which compared those with and without diabetes, the presence of diabetes was reported to incur significant additional hospitalization costs of $32,577 versus no diabetes $29,150 [10]. Although evidence from such studies helps to clarify how resource use varies for patients with differing clinical presentations, the implications for policy are limited insofar as they reinforce the well-acknowledged relationship between more severe and complex illness and higher healthcare costs.

Investigation into the broader socioeconomic and regional health systems factors that influence costs is generally limited. Notably, however, a study in India using national administrative and household survey data reported that costs of hospitalization for cardiovascular disease were significantly higher in private health centers, in patients of high-fertility status, and in those of high socioeconomic status [11]. Studies of this kind, which examine broader healthcare systems and socioeconomic drivers of healthcare costs, provide potential policy lessons by addressing disparities in costs that reflect possible inefficiencies in healthcare systems, and are thus amenable to policy intervention.

In this study we assessed hospitalization costs associated with treating ACS patients across eight countries/regions in Asia: China, Hong Kong, India, Malaysia, Singapore, South Korea, Thailand, and Vietnam. The aim was to highlight variations in care costs across different healthcare settings and by different categories of ACS (ST-elevation myocardial infarction [STEMI], non-STEMI [NSTEMI] and unstable angina [UA]), and to determine the clinical, socioeconomic, and healthcare system factors that predict whether patients incur a level of treatment cost equivalent to the highest quintile by country and type of ACS.

## Methods

The EPICOR Asia (long-tErm follow-uP of antithrombotic management patterns In acute CORonary syndrome patients in Asia) study (NCT01361386; registration, May 26, 2011), was a prospective, multinational, observational, cohort study of patients hospitalized for ACS enrolled in 218 hospitals in eight countries/regions in Asia [12]. These countries represent a combination of high- (Singapore, Hong Kong, Republic of Korea), upper-middle (China, India, Malaysia), and lower-middle (India, Vietnam) income settings. EPICOR Asia recruited consecutive patients hospitalized for ACS within 48 h of symptom onset and who were discharged with a final diagnosis of STEMI, NSTEMI, or UA, with 2-yearfollow-up. Data collection occurred between 2011 and 2014.

The study was conducted in compliance with the principles of the Declaration of Helsinki, International Conference on Harmonization Good Clinical Practice guidelines and applicable legislation on non-interventional studies in participating countries and regions. The protocol, including the informed consent form, was approved in writing by the applicable ethics committee of the participating centers in accordance with local regulations in each country. The ethics committee also approved any other non-interventional study documents in accordance with local regulations. A list of participating centers is provided in Additional file 1: Table S1. Patients provided written informed consent at discharge and completed a contact order form agreeing to be contacted for regular follow-up interviews post-discharge.

Data were collected from 12,922 patients on demographics, medical history, event characteristics, and socioeconomic and insurance status at discharge. Patients were followed up at 6 weeks following hospitalization for the index event in regard to treatment course. Costs associated with hospitalization were estimated from a health sector perspective based on health system payments for individual items at each specific hospital. Such payments reflect the outlay required of payers and are therefore of primary relevance from a policy standpoint. Costs were converted into US dollars at the prevailing exchange rates (see footnote to Table 2). These have not been converted into international dollars although prevailing purchasing power parities (PPPs) are presented to enable the reader to make such a conversion. In spite of potentially skewed distributions, mean costs were reported in accordance with economic theory which deems that the arithmetic mean (unlike the median) best informs resource allocation given a budgetary constraint [13–17].

The primary outcome was whether a patient incurred costs in the highest quintile for their specific country and index event diagnosis. This type of binary outcome, standardized against country and condition-specific norms, facilitated the pooling of data from multiple countries with differences in living standards and cost structures.

We assessed the association between the outcomes variable and several demographic, socioeconomic, health and clinical systems variables through univariate analyses. These variables included age, sex, smoking status, income (defined by country-specific quintiles based on country- specific income distributions, with quintile 1 representing the lowest income group, and quintile 5 the highest), country, place of residence (rural versus metropolitan), insurance status, cardiovascular disease history, hospitalization in the 3 months prior to the index event, dependence before the index event, index event medical management (invasive, non-invasive or unknown), type of hospital (regional/community/rural, non-university general hospital, university general hospital, other type of hospital/clinic), number of beds within the facility, and length of stay. A multivariable logistic regression model was constructed using stepwise selection, forcing in a variable of interest – health insurance status. A conditional binary regression model [18] to conduct a matched analysis was not used because the objective in this study was not to estimate a treatment effect.

Analyses were undertaken using SAS® v8.2 or later (SAS Institute, Cary, USA).

## Results

Cost data were available for 10,819 participants; data from Malaysia (*n* = 42 patients) were excluded as costs were incorrectly recorded. Overall, 71% of participants were from China and 21% from India. Hong Kong, South Korea, Thailand, and Vietnam comprised between 1.2–2.3% of participants, with Singapore representing the fewest participants (0.6%).

Participant mean age was 60 years, 77% were males and 33% were current smokers compared with 20% former smokers, and 40% who had never smoked. Most participants were concentrated in the second (42%) and third (25%) income quintiles. Quintile 1 comprised less than 1% of participants, quintile 4 had 2% and quintile 5 had 24% (Table 1).

**Table 1:** Baseline characteristics by final diagnosis of index event – all patients with cost data

	STEMI (*n* = 5478)	NSTEMI (*n* = 2030)	UA (*n* = 3311)	Total (*n* = 10,819)
Age, mean (SD)	58.5 (11.7)	61.9 (11.95)	61.4 (10.44)	60.0 (11.48)
Male, n (%)	4532 (82.7)	1520 (74.9)	2247 (67.9)	8299 (76.7)
Smoker, n (%)				
Current	2197 (40.1)	649 (32.0)	742 (22.4)	3588 (33.2)
Former	964 (17.6)	398 (19.6)	775 (23.4)	2137 (19.8)
Never	1896 (34.6)	855 (42.1)	1602 (48.4)	4353 (40.2)
Unknown	421 (7.7)	128 (6.3)	192 (5.8)	741 (6.8)
Income, n (%)				
Quintile 1	14 (0.3)	21 (1.0)	18 (0.5)	53 (0.5)
Quintile 2	2352 (42.9)	849 (41.8)	1342 (40.5)	4543 (42.0)
Quintile 3	1253 (22.9)	471 (23.2)	965 (29.1)	2689 (24.9)
Quintile 4	70 (1.3)	61 (3.0)	31 (0.9)	162 (1.5)
Quintile 5	1271 (23.2)	463 (22.8)	805 (24.3)	2539 (23.5)
Country, n (%)				
China	3716 (67.8)	1234 (60.8)	2754 (83.2)	7704 (71.2)
Hong Kong	75 (1.4)	45 (2.2)	5 (0.2)	125 (1.2)
India	1341 (24.5)	527 (26.0)	415 (12.5)	2283 (21.1)
Singapore	25 (0.5)	36 (1.8)	4 (0.1)	65 (0.6)
South Korea	101 (1.8)	70 (3.4)	76 (2.3)	247 (2.3)
Thailand	124 (2.3)	81 (4.0)	30 (0.9)	235 (2.2)
Vietnam	96 (1.8)	37 (1.8)	27 (0.8)	160 (1.5)
Place of residence, n (%)				
Rural	2088 (38.1)	624 (30.7)	1067 (32.2)	3779 (34.9)
Metropolitan	3390 (61.9)	1406 (69.3)	2244 (67.8)	7040 (65.1)
Insurance status, n (%)				
Yes	4396 (80.2)	1620 (79.8)	2950 (89.1)	8966 (82.9)
No	1082 (19.8)	410 (20.2)	361 (10.9)	1853 (17.1)
Disease history, n (%)	1008 (18.4)	630 (31.0)	1437 (43.4)	3075 (28.4)
Myocardial infarction	354 (6.5)	247 (12.2)	421 (12.7)	1022 (9.4)
Prior PCI	201 (3.7)	173 (8.5)	455 (13.7)	829 (7.7)
Prior CABG	43 (0.8)	39 (1.9)	70 (2.1)	152 (1.4)
CAG diagnostic for CAD	233 (4.3)	216 (10.6)	606 (18.3)	1055 (9.8)
Chronic angina	484 (8.8)	299 (14.7)	1018 (30.7)	1801 (16.6)
Heart failure	63 (1.2)	71 (3.5)	135 (4.1)	269 (2.5)
Atrial fibrillation	47 (0.9)	45 (2.2)	62 (1.9)	154 (1.4)
TIA/stroke	212 (3.9)	101 (5.0)	166 (5.0)	479 (4.4)
Peripheral vascular disease	24 (0.4)	24 (1.2)	41 (1.2)	89 (0.8)
Chronic renal failure	59 (1.1)	70 (3.4)	38 (1.1)	166 (1.5)
Hospitalization in 3 months prior to index event, n (%)	206 (3.8)	126 (6.2)	458 (13.8)	790 (7.3)
Dependence degree (need of help for daily activities) prior to index event, n (%)				
Some dependence	715 (13.1)	318 (15.7)	367 (11.1)	1400 (12.9)
No dependence	4601 (84.0)	1659 (81.7)	2826 (85.4)	9086 (84.0)
Unknown	162 (3.0)	53 (2.6)	118 (3.6)	333 (3.1)
Index event medical management, n (%)				
Invasive	4713 (86.0)	1598 (78.7)	2612 (78.9)	8923 (82.5)
Non-invasive	724 (13.2)	422 (20.8)	617 (18.6)	1763 (16.3)
Unknown	41 (0.7)	10 (0.5)	82 (2.5)	133 (1.2)
Type of hospital, n (%)				
Regional/community/rural hospital	296 (5.4)	111 (5.5)	76 (2.3)	483 (4.5)
Non-university general hospital	1269 (23.2)	368 (18.1)	792 (23.9)	2429 (22.5)
University general hospital	2931 (53.5)	1074 (52.9)	2092 (63.2)	6097 (56.4)
Other type of hospital/clinic	982 (17.9)	477 (23.5)	351 (10.6)	1810 (16.7)
Number of beds, mean (95% CI)	1307.1 (1280.0, 1334.2)	1223.1 (1181.9, 1264.3)	1374.5 (1338.6, 1410.5)	1311.9 (1292.7, 1331.2)
Length of stay, mean (95% CI)	10.3 (10.2, 10.5)	10.2 (9.9, 10.6)	9.8 (9.6, 10.0)	10.1 (10.0, 10.3)

*CABG* Coronary artery bypass graft, *CAD* Coronary artery disease, *CAG* Coronary angiogram, *CI* Confidence interval, *NSTEMI* Non-ST-elevation myocardial infarction, *PCI* Percutaneous coronary intervention, *STEMI* ST-elevation myocardial infarction, *TIA* Transient ischemic attack*, UA* Unstable angina

The majority (65%) of participants were resident in metropolitan areas compared with 35% in rural areas; 83% of participants had health insurance; 28% had some cardiovascular disease history; 7% had been hospitalized in the 3 months before the index event; 13% had some degree of dependence before the index event; and 83% underwent an invasive procedure during hospitalization. In terms of hospital type, 5% were admitted to a regional/community/rural hospital, 23% to a non-university general hospital, 56% to a university general hospital, and 17% to a ‘other’ type of hospital or clinic. The mean number of beds was 1312, and mean length of stay was 10.1 days, with little difference between STEMI (10.3 days), NSTEMI (10.2 days) and UA (9.8 days) (Table 1).

**Fig. 1:**
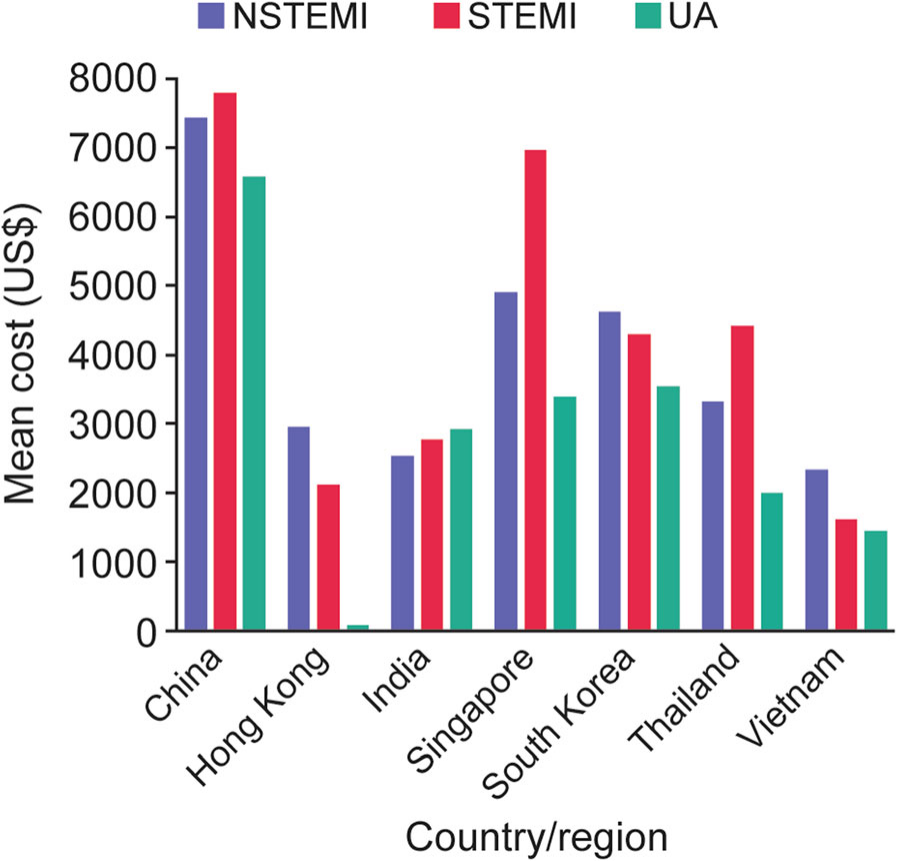
Mean cost (US$) by country and index event. *NSTEMI* non-ST-elevation myocardial infarction, *STEMI* ST-elevation myocardial infarction, *UA* unstable angina

When compared in US dollars, the highest-cost countries were China (STEMI mean cost = $7790; NSTEMI = $7450; UA = $6585), Singapore ($6978; $4910; $3394), the Republic of Korea ($4300; $4621; $3552), and Thailand ($4427; $3321; $2008); across the three index-event types, UA generally represented the lowest-cost category except in India where it was the highest, albeit modestly (Fig. 1). In Hong Kong, all emergency admissions for ACS are subsidised by the Government and although the real cost incurred is not clear, health system payments to the patient are generally low. In terms of invasive interventional procedures, the highest costs were associated with (in descending order) coronary artery bypass graft, percutaneous coronary intervention (PCI) with one drug-eluting stent, PCI with one bare metal stent, and angiography; the costs for these procedures being highest in China, Hong Kong, Singapore, and Thailand (Table 2; also, conversion of these costs based on purchasing power parities (PPP) into international dollars is provided in Additional file 1: Table S2). Univariate analyses indicated that high-cost utilization was significantly associated with income, hospitalization in the 3 months prior to the index event, degree of dependence before the index event, index-event medical management, length of stay, sex, type of hospital and disease history (Table 3).

**Table 2:** Mean (95% confidence interval [CI]) individual center-specific cost ($) per procedure by country^a^

	Country/Region							
	China (*n* = 7704)	Hong Kong (*n* = 125)	India (*n* = 2283)	Singapore (*n* = 65)	South Korea (*n* = 247)	Thailand (*n* = 235)	Vietnam (*n* = 160)	All (*n* = 10,819)
ECG	4.2 (3.9, 4.4)	43.7 (38.1, 49.3)	3.2 (2.8, 3.6)	35.0 (NA)	7.6 (5.5, 9.7)	8.8 (6.6, 11.0)	1.2 (0.7, 1.7)	5.9 (4.9, 6.8)
Cardiac markers	32.9 (30.2, 35.5)	51.5 (41.8, 61.1)	26.7 (23.0, 30.4)	84.0 (NA)	30.3 (22.6, 38.0)	15.5 (11.9, 19.0)	7.8 (0.9, 14.6)	35.8 (22.5, 49.1)
Echocardiography	53.9 (42.5, 65.3)	261.6 (226.9, 296.3)	25.2 (22.1, 28.3)	443.0 (NA)	184.9 (167.5, 202.3)	72.4 (65.7, 79.0)	9.3 (5.7, 12.9)	69.5 (59.3, 79.6)
Angiography	666.4 (613.1, 719.6)	1536.0 (816.9, 2255.1)	343.8 (203.6, 484.0)	4674.0 (NA)	667.0 (376.0, 958.0)	645.2 (539.3, 751.1)	284.2 (209.8, 358.6)	621.4 (549.6, 693.3)
PCI with 1 drug eluting stent	3072.8 (2820.6, 3325.1)	4359.2 (2615.1, 6103.3)	3696.7 (2251.7, 5141.8)	17,590.0 (NA)	1980.9 (1464.5, 2497.4)	4261.4 (3456.8, 5066.1)	3233.3 (2729.3, 2737.4)	3308.1 (2918.5, 3697.7)
PCI with 1 bare metal stent	2174.9 (1924.9, 2425.0)	1942.4 (617.2, 3715.6)	2192.2 (1858.5, 2525.9)	7995.0 (NA)	1752.4 (1291.4, 2213.4)	3247.6 (2609.0, 3886.1)	1900.0 (1542.6, 2257.4)	2251.1 (2067.0, 2435.3)
CABG	8864.1 (8173.1, 9555.1)	7561.0 (4368.2, 10,753.8)	3265.1 (2918.4, 3611.7)	29,520.0 (NA)	4458.6 (2879.3, 6038.0)	7240.7 (4485.7, 9995.7)	4160.0 (2980.1, 5339.9)	6906.7 (6284.3, 7529.0)
Stay in CCU per day	164.5 (119.7, 209.2)	1482.0 (1233.7, 1730.3)	90.7 (72.5, 108.8)	861.0 (NA)	123.2 (73.4, 173.0)	109.7 (59.3, 160.0)	35.3 (4.4, 72.3)	231.3 (102.1, 360.5)

*CABG* Coronary artery bypass graft, *CCU* Critical care unit, *ECG* Electrocardiogram, *NA* Not available, *PCI* Percutaneous coronary intervention

^a^Exchange rate conversion based on (March 2013): 1 USD = 6.2943 CNY; 7.7681 HKD; 53.060 INR; 3.1690 SGD; 1158.1 KRW; 31.545 THB; 21,030 VND

**Table 3:** Model-based point estimates for high-cost healthcare expenditure^a^ using logistic models – univariate analysis (excluding Malaysia)

Factor	Odds ratio	95% CI	*P* value
Age, per 10-year increment	1.04	0.99, 1.08	0.0925
Sex, male versus female	1.18	1.06, 1.33	0.0038
Income (versus quintile 5)			< 0.0001
Quintile 1	0.45	0.19, 1.06	
Quintile 2	0.76	0.67, 0.85	
Quintile 3	0.97	0.85, 1.11	
Quintile 4	0.80	0.53, 1.20	
Health insurance, yes versus no	1.02	0.90, 1.16	0.7074
Residence, rural versus non-rural	1.02	0.92, 1.12	0.7516
Smoker (versus never)			0.2418
Current	0.92	0.83, 1.03	
Former	1.02	0.89, 1.15	
Disease history, yes versus no	1.15	1.04, 1.28	0.0068
Hospitalization in the 3 months prior to index event, yes versus no	1.37	1.16, 1.62	0.0002
Dependence degree before index event, none versus some	1.74	1.48, 2.05	< 0.0001
Index event medical management, invasive versus non-invasive	4.62	3.78, 5.64	< 0.0001
Type of hospital (versus UGH)			0.0030
Regional/community/rural hospital	0.92	0.72, 1.17	
Non-UGH	1.13	1.01, 1.27	
Other type of hospital/clinic	1.24	1.09, 1.40	
Number of beds	1.00	1.00, 1.00	0.5995
Length of stay	1.04	1.03, 1.05	< 0.0001
Country (versus China)			0.8569
Hong Kong	1.00	0.64, 1.56	
India	1.09	0.97, 1.23	
Singapore	0.91	0.48, 1.70	
South Korea	1.00	0.72, 1.36	
Thailand	1.00	0.72, 1.38	
Vietnam	0.96	0.65, 1.43	

*UGH* University general hospital, CI Confidence interval

^a^high cost defined as the top quintile within a country

In controlling for all these variables at once, a multivariate analysis indicates the significant predictors of high-cost care were age (odds ratio [OR] = 1.10 per 10-year increment), being male (OR = 1.17), income (quintile 2 versus 5: OR = 0.76), prior disease history (OR = 1.25), hospitalization in the 3 months prior to index event (OR = 1.48), no dependency prior to index event (OR = 1.96), having an invasive procedure (OR = 5.48), hospital type (community versus university general: OR = 1.74), country (Hong Kong versus China: OR = 1.92; India versus China: OR = 2.54; Thailand versus China: OR = 1.52) and length of stay (OR = 1.06 per day) (Table 4).

**Table 4:** Model-based point estimates for high-cost healthcare expenditure^a^ using logistic models – multivariate analysis (excluding Malaysia)

Factor	Odds ratio	95% CI	*P* value
Age, per 10-year increment	1.10	1.05, 1.16	< 0.0001
Sex, male versus female	1.17	1.02, 1.33	0.0224
Income (versus quintile 5)			< 0.0001
Quintile 1	0.43	0.15, 1.19	
Quintile 2	0.76	0.67, 0.86	
Quintile 3	0.96	0.83, 1.11	
Quintile 4	0.74	0.45, 1.23	
Health insurance, yes versus no	1.11	0.89, 1.38	0.3686
Disease history, yes versus no	1.25	1.11, 1.41	0.0002
Hospitalization in the 3 months prior to index event, yes versus no	1.48	1.23, 1.77	< 0.0001
Dependence degree before index event, none versus some	1.96	1.60, 2.40	< 0.0001
Index event medical management, invasive versus non-invasive	5.48	4.34, 6.92	< 0.0001
Type of hospital (versus UGH)			0.0016
Regional/community/rural hospital	1.74	1.25, 2.41	
Non-UGH	1.13	0.99, 1.29	
Other type of hospital/clinic	0.97	0.79, 1.19	
Length of stay	1.06	1.04, 1.06	< 0.0001
Country (versus China)			< 0.0001
Hong Kong	1.92	1.04, 3.53	
India	2.54	1.98, 3.25	
Singapore	1.58	0.77, 3.24	
South Korea	1.21	0.76, 1.95	
Thailand	1.52	1.05, 2.20	
Vietnam	0.90	0.57, 1.42	

*UGH* University general hospital

^a^Defined as the top quintile within a country

## Discussion

This study in more than 10,000 participants represents one of the largest prospective cost analyses of ACS and one of only few such analyses to provide cross-country comparisons. We found substantial variations across countries/regions and index diagnosis in healthcare costs incurred by patients during hospitalization for treatment of an ACS event. This is perhaps to be expected given the milieu of high, upper-middle and lower-middle income countries included in EPICOR Asia. What seems surprising is that the cost of treating ACS appeared relatively high in China across all three index-event types, exceeding those recorded for high-income countries/regions such as Singapore, the Republic of Korea, and Hong Kong. Interestingly, in contrast to Hong Kong and Singapore, costs in China were generally similar across all three index-event types. This suggests stratification of patients may not have been optimal, with patients at high- and lower-risk variably receiving high-level interventional therapy, and of variable cost. This may be compounded by inaccurate recording as to whether percutaneous transluminal coronary angioplasty was provided with or without stenting. Further detailed study is required to establish the multifarious factors underlying the apparent high costs of treatment in China and alleviate any concerns to decision makers given the increasing burden of ACS and growing proliferation of treatment. For example, between 2007 and 2011, there was a virtual doubling in the number of PCI procedures performed from 180,000 to 330,000 [19].

The finding that age is a positive predictor of high cost is consistent with potentially greater complexity and severity of illness, some of which may not have been captured and thus controlled for in the model. This is perhaps further evidenced by the positive association between hospitalization in the 3 months prior to the index event and high costs. Similar findings for male sex are consistent with a study in Italy where costs incurred by men were significantly greater than that for women, irrespective of index-event type [8]. Reports that women who present with ACS may be evaluated less intensively than men, may go some way towards explaining this [20].

The findings reported here also provide evidence of a potential income effect in that patients on relatively high income appear more likely to be categorized as highest cost. Odds ratios relative to income quintile 5 of 0.45 for quintile 1; 0.76 for quintile 2; 0.97 for quintile 3; 0.80 for quintile 4 (although only statistically significant in relation to quintile 2), ostensibly indicate a pattern in which the odds of incurring high costs increase with income. Such a finding, again, accords with expectations that wealthier patients will seek and have access to higher-cost treatments.

The findings that longer length of stay and having an invasive procedure (versus non-invasive medical management) were both positively associated with odds of incurring high costs is consistent with expectations and reflects, perhaps obviously, resource needs associated with longer treatment duration and need for an invasive procedure. Less intuitive is the finding that those patients who required no help with daily activities prior to hospitalization for their index condition (“no dependence”) had significantly higher odds of being in the highest-cost category. Here, it is possible that patients with dependency at baseline would have the ongoing support of a “carer” to rely on. The potential lack of such support for patients without dependency at baseline may have led to greater costs due to a greater need for in-hospital rehabilitation and extensive discharge planning.

Another ostensibly unexpected observation was that the odds of incurring high-cost treatment, relative to those encountered in patients admitted to a university general hospital, were higher for those patients admitted to all “other” categories of hospital, e.g. regional/community/rural hospitals, non-university general hospitals and other type of clinics. Here, it is possible that the multivariable analysis used in this study effectively controlled for factors implicated in higher costs seen in teaching (university) hospitals, such as size of facility, treatment mode, disease history and length of stay. Our findings suggest, therefore, that the independent effect of university status of a hospital was to lower costs, very likely associated with efficiency and an established degree of expertise in such centers.

The lack of significant association between insurance status and high-cost care may allay potential concerns about the inflationary effects of national programs to expand insurance coverage, e.g. due for instance to incentives created by a third-party payer for providers to overcharge/over-service (provider moral hazard) and patients to overuse (patient moral hazard) [21]. Although this study focuses only on ACS patients, the findings of this study found no evidence of an inflationary impact associated insurance coverage. Further country-specific research is needed to determine whether the roll out of social insurance programs will increase costs to any significant degree.

There were several limitations in the present study. First, inclusion only of patients alive and followed up at 6 weeks might suggest a possible survivor bias to the findings. As mentioned, earlier studies have reported in-hospital mortality to be associated with higher costs, suggesting our estimates of average costs may have been underestimated. In addition, the costs examined in this study reflect only health system cost whereas a broader societal perspective would have considered costs to households and the community associated with indirect loss of income and reduced productivity. Also, the costs included in this analysis were confined to hospitalization for the index condition and excluded costs of potential re-hospitalizations for ACS; in the US, such costs have been estimated at over 30% [22], suggesting there are significant costs associated with ACS outside of the scope of this analysis. Furthermore, the costs of sub-acute follow-up care were not included. These may vary across countries due to differences in treatment norms, funding models and other health system characteristics. Despite these potential limitations in capturing the high costs to health systems associated with ACS, the study highlights the major policy challenges associated with a high burden of illness in Asia. Some countries in this analysis were represented by a relatively small number of participants, thus precluding detailed country-specific analyses. Thus, the way in which the primary outcome for this study was specified (i.e. occurrence of cost in the highest quintile specific to each country and index condition) served as a standardized outcome that facilitated the pooling of data across all countries. An alternative approach would have been to adjust for differences in purchasing power by converting into international dollars; however, the problem with such a strategy is that costs reported in international dollars lack meaning for local policy makers since they do not reflect actual budgetary implications (nevertheless the conversions are provided in Additional file 1: Table S2 for reference). The inclusion of hospital length of stay as an explanatory variable and the likelihood of it being highly correlated with cost is a potential weakness in the modelling [23], as we may not be able to identify factors that affect the cost through the hospital length of stay. However, it is an important variable of interest and its inclusion is justified as it allows us to estimate the direct effect of other factors included in the model. Finally, without accounting for clustering in the analysis, variance and confidence intervals could be slightly underestimated. However, in international studies of this kind, it is conventional that such adjustments are not made.

## Conclusion

The present analysis highlights the drivers of high-cost treatment for ACS in Asia. It represents an advance in this area by examining factors beyond the clinical drivers of costs. The study further identifies health-system factors including hospital type, and health insurance and socioeconomic status, providing evidence to policy makers of the financial implications of current and future reforms; notably programs in Asia to expand health insurance coverage to underserved populations. The value of prevention programs in avoiding hospitalizations for ACS is also considered to highlight population groups (e.g. men, high-income groups, and uninsured) in whom effective prevention may yield the greatest financial savings.

## Additional file

Additional file 1: **Table S1.** List of participating sites and principal investigators. **Table S2.** Mean (95% CI) individual center-specific cost ($INT) per procedure by country*. (DOCX 34 kb)

## Abbreviations

ACS: Acute coronary syndromes
CABG: Coronary artery bypass graft
CAD: Coronary artery disease
CAG: Coronary angiogram
CCU: Critical care unit
CI: Confidence interval
ECG: Electrocardiogram
NA: Not available
NSTEMI: Non-ST-elevation myocardial infarction
OR: Odds ratio
PCI: Percutaneous coronary intervention
STEMI: ST-elevation myocardial infarction
TIA: Transient ischemic attack
UA: Unstable angina
UGH: University general hospital
