# Supplementary material for: Predictors of high-cost hospitalization in the treatment of acute coronary syndrome in Asia: findings from EPICOR Asia
Source: BMC Cardiovasc Disord. 2018 Jul 4;18:139. doi: 10.1186/s12872-018-0859-4 (PMC6033225; doi:10.1186/s12872-018-0859-4)
Supplement: Supplementary file 1 — Table S1. List of participating sites and principal investigators. Table S2. Mean (95% CI) individual center-specific cost ($INT) per procedure by country*. (DOCX 34 kb) [file 12872_2018_859_MOESM1_ESM.docx]

**Supplementary materials**

**Supplementary Table SI. List of participating sites and principal investigators**

| **Country** | **Site number** | **Site name** | | **Investigator name** |
| --- | --- | --- | --- | --- |
| China | 001 | Peking University First Hospital | | Huo Yong |
| China | 002 | Beijing An Zhen Hospital | | Lv Shu Zheng |
| China | 003 | Armed Police General Hospital | | Liu Hui Liang |
| China | 004 | Peking University People’s Hospital | | Wang Wei Ming |
| China | 005 | Wuxi 2nd People’s Hospital | | Jin Yan |
| China | 006 | The Chinese PLA General Hospital | | Chen Yun Dai |
| China | 007 | The Central Hospital of China Aerospace Industry Corporation | | Wang Bin |
| China | 008 | Beijing Shi Jing Shan Hospital | | Wang Ming Sheng |
| China | 009 | Beijing Hospital of Traditional Chinese Medicine Affiliated to Capital Medical University | | Liu Hong Xu |
| China | 010 | Beijing Xuan Wu Hospital Affiliated to Capital Medical University | | Xu Dong |
| China | 011 | Beijing Tong Ren Hospital Affiliated to Capital Medical University | | Wu Ming Ying |
| China | 012 | Fuwai Cardiovascular Hospital | | Qiao Shu Bing |
| China | 013 | The 4th Hospital Affiliated to China Medical University | | Jin Yuan Zhe |
| China | 015 | The General Hospital of Shenyang Military Region | | Han Ya Ling |
| China | 016 | People’s Hospital of Liao Ning Province | | Li Zhan Quan |
| China | 017 | Central Hospital of Yingkou City | | Sun Hong Tai |
| China | 018 | The People’s Hospital, He Bei Province | | Qi Xiao Yong |
| China | 019 | The 2nd hospital of He Bei Medical University | | Liu Fan |
| China | 020 | Chinese PLA Bethune International Peace Hospital | | Wang Dong Mei |
| China | 021 | Chinese PLA 252th Hospital | | Cao Xue Bin |
| China | 022 | Tangshan Gong Ren Hospital | | Liu Xiao Kun |
| China | 023 | | Changzhi Heping Hospital | Wang Zhi Ping |
| China | 024 | | Jinzhong First People's Hospital | Yan Yu Hu |
| China | 025 | | Xishan Coal Corporation Staff General Hospital | Zhang Qiao Ling |
| China | 026 | | The 2nd hospital Affiliated to Shan Xi Medical University | Wang Feng Zhi |
| China | 027 | | Cardiovascular Hospital of Shan Xi Province | Li Bao |
| China | 028 | | General Hospital of Lu An Group | Wei Hai Song |
| China | 029 | | 1st hospital of Liao Ning Medical College | Tao Gui Zhou |
| China | 030 | | Teda International Cardiovascular Hospital | Qi Xiang Qian |
| China | 031 | | 2nd hospital affiliated to Harbin Medical University | Yu Bo |
| China | 032 | | Tianjin Chest Hospital | Cong Hong Liang |
| China | 033 | | Tianjin Armed Police Hospital | Jiang Tie Min |
| China | 034 | | Tianjin Union Medicine Center | Yao Zhu Hua |
| China | 036 | | General Hospital of Da Qing Oil Group | Li Hui |
| China | 037 | | The First Hospital of Jilin University | Zheng Yang |
| China | 038 | | 2nd Hospital of Jilin University | Liu Bin |
| China | 040 | | The Affiliated Hospital of Guiyang Medical College | Wu Li Rong |
| China | 041 | | Sichuan Provincial People’s Hospital | Tao Jian Hong |
| China | 042 | | 1st People's Hospital of Yunnan Province | Zhang Hong |
| China | 043 | | 2nd People's Hospital of Yunnan Province | Li Jian Mei |
| China | 044 | | 1st Hospital Affiliated to Kunming Medical College | Guo Tao |
| China | 045 | | Kunming General Hospital of Chengdu Military Region | Yang Li Xia |
| China | 046 | | The 1st People's Hospital of Yulin City | Li Ping |
| China | 047 | | People’s Hospital of Guangxi Province | Lin Ying Zhong |
| China | 048 | | 1st Hospital Affiliated to Guangxi Medical University | Li Lang |
| China | 049 | | The 1st Affiliated Hospital, Sun Yat-Sen University | Du Zhi Min |
| China | 050 | | General hospital of Guangzhou Military Command of PLA | Qiu Jian |
| China | 051 | | Guangzhou Red Cross Hospital | Wu Tong Guo |
| China | 052 | | The 2nd hospital affiliated to Guangzhou Medical University | Xiong Long Gen |
| China | 053 | | Nanfang Hospital affiliated to Southern Medical University | Hou Yu Qing |
| China | 054 | | Guangdong General Hospital | Chen Ji Yan |
| China | 055 | | Shenzhen People’s Hospital | Dong Shao Hong |
| China | 056 | | Peking University Shenzhen Hospital | Wu Chun |
| China | 057 | | Shenzhen SUN YAT-SEN Cardiovascular Hospital | Peng Chang Nong |
| China | 058 | | The 1st people’s hospital of Foshan City | Yang Xi Li |
| China | 059 | | Tongji Hospital of Tongji Medical College, Huazhong University of Science and Technology | Wang Dao Wen |
| China | 060 | | Wu Han Puai Hospital | Gu Ye |
| China | 061 | | Wuhan Asia Heart Hospital | Su Xi |
| China | 062 | | Taihe Hospital of Shiyan | Wang Chong Quan |
| China | 063 | | Xiangya Hospital of Central South University | Yang Tian Lun |
| China | 064 | | The Second Xiangya Hospital of Central South University | Hu Xinqun |
| China | 065 | | 3rd Xiangya Hospital of Central South University | Yang Tian Lun |
| China | 067 | | The First Affiliated Hospital of Henan University of Science and Technology | Dong Ping Shuan |
| China | 068 | | The 1st Hospital affiliated to Fujian Medical University | Lin Jin Xiu |
| China | 069 | | Hangzhou Hospital of Traditional Chinese Medicine | Chen Tie Long |
| China | 070 | | Sir Run Run Shaw Hospital Affiliated to School of Medicine Zhe Jiang University | Fu Guo Sheng |
| China | 071 | | Jinan Central Hospital affiliated to Shandong University | Su Guo Hai |
| China | 072 | | Qi Lu Hospital affiliated to Shandong University | Chen Yu Guo |
| China | 073 | | Shan Dong Province Qian Fo Shan Hospital | Hou Ying Long |
| China | 074 | | Anhui Provincial Hospital | Yan Ji |
| China | 075 | | Yijishan Hospital affiliated to Wannan Medical College | Cao Heng |
| China | 076 | | Nanjing Red Cross Hospital | Pei Su Li |
| China | 077 | | Zhongda Hospital affiliated to Southeast University | Ma Gen Shan |
| China | 078 | | Jiangsu Hospital of Traditional Chinese Medicine | Wang Zhen Xing |
| China | 079 | | The 2nd hospital affiliated to Nanjing Medical University | Lu Xiang |
| China | 080 | | Xuzhou City Center Hospital | Fu Qiang |
| China | 081 | | Zhongshan Hospital affiliated to Fu Dan University | Ge Jun Bo |
| China | 082 | | 10th hospital of Tongji University | Xu Ya Wei |
| China | 083 | | Shanghai Chest Hospital | Fang Wei Yi |
| China | 084 | | Rui Jin Hospital of Shanghai Jiao Tong University | Zhang Rui Yan |
| China | 085 | | Ren Ji Hospital of Shanghai Jiao Tong University, School of Medicine | He Ben |
| China | 086 | | Shanghai Changhai Hospital | Qin Yong Wen |
| China | 087 | | Qingdao Municipal Hospital | Wang Xu |
| China | 088 | | The affiliate hospital of medical college, Qingdao University | Cai Shang Lang |
| China | 090 | | Wu Xi People's Hospital | Yang Zhen Yu |
| China | 091 | | Zhenjiang Jiangbin Hospital | Yan Jin Chuan |
| China | 092 | | The 1st hospital affiliated to Chongqing Medical University | Lei Han |
| China | 093 | | The First Affiliated Hospital of Third Military Medical University | Song Zhi Yuan |
| China | 094 | | The 2nd hospital affiliated to Third Military Medical University | Huang Lan |
| China | 095 | | The Third Affiliated Hospital of Third Military Medical University | Chunyu Zeng |
| China | 096 | | Shihezi University First Affiliated Hospital | Qiu Qiang |
| China | 097 | | Traditional Chinese Medicine Hospital of Xinjiang Uygur Autonomous Region | Wu Gang |
| China | 098 | | 1st hospital affiliated to Xinjiang Medical University | Ma Yi Tong |
| China | 099 | | General Hospital of Chengdu Military Region of PLA | Yang Yong Jian |
| China | 100 | | West China Hospital, Si Chuan University | Xu Yuan Ning |
| China | 101 | | First Affiliated Hospital of Xi'an Jiao Tong University, College of Medicine | Wang Yan Ni |
| China | 102 | | Shaanxi Provincial People's Hospital | Wang Jun Kui |
| China | 103 | | The 1st Hospital affiliated to Lanzhou University | Zhang Zheng |
| China | 104 | | The 2nd Hospital affiliated to Lanzhou University | Bai Feng |
| China | 105 | | General Hospital of Lanzhou Military Region | Zhang Wei Ze |
| China | 106 | | Qinghai University Affiliated Hospital | Liu Yin |
| China | 107 | | Qinghai People’s Hospital | Zhou Bai Li |
| China | 108 | | General Hospital of Ningxia Medical University | Xue Li |
| China | 109 | | People’s Hospital, Henan Province | Gao Chuan Yu |
| China | 110 | | Zhengzhou Central Hospital | Zhao Zhi Chen |
| China | 111 | | The 1st Hospital affiliated to Zhengzhou University | Qiu Chun Guang |
| China | 112 | | 1st affiliated hospital of Henan College of Traditional Chinese Medicine | Guan Huai Min |
| Hong Kong | 001 | | Queen Mary Hospital | Stephen W-L Lee |
| Hong Kong | 002 | | Prince of Wales Hospital | Lam Yat Yin |
| Hong Kong | 003 | | Queen Elizabeth Hospital | Chan Kam Tim |
| Hong Kong | 004 | | Princess Margaret Hospital | Tsui Ping Tim |
| Hong Kong | 005 | | Yan Chai Hospital | Tam Kin Ming |
| India | 001 | | Sir Ganga Ram Hospital | J P S Sawhney |
| India | 002 | | Fortis Escorts Heart Institute, New Delhi | Upendra Kaul |
| India | 004 | | Maharaja Agarsen Hospital, New Delhi | B B Chanana |
| India | 006 | | Artemis Hospital, Gurgaon | Manjinder Singh Sandhu |
| India | 007 | | Paras Hospital, Gurgaon | Sanjeev Chaudhary |
| India | 008 | | Peerless Hospital, Kolkata | S K Paul |
| India | 009 | | B M Birla Heart & Research Centre, Kolkata | Dhiman Kahali |
| India | 010 | | Fortis Hospital, Mohali | H K Bali |
| India | 011 | | Kulwant Heart Centre | Rakesh Kumar Aggarwal |
| India | 012 | | Medicity Hospital, Ludhiana | Ashish Saxena |
| India | 013 | | Heart & General Hospital, Jaipur | Prakash Chandwani |
| India | 014 | | Fortis Hospital, Jaipur | Rajeev Gupta |
| India | 015 | | Holy Family Hospital, Mumbai | Brian Pinto |
| India | 016 | | Kokilaben Hospital, Mumbai | J J Dalal |
| India | 017 | | K E M Hospital, Mumbai | Prafulla Kerkar |
| India | 018 | | P D Hinduja Hospital, Mumbai | Navneet Kumar |
| India | 019 | | Ruby Hall Clinic, Pune | Shirish Hiremath |
| India | 020 | | Deenanath Mangeshkar Hospital and Research Center, Mumbai | Shireesh Sathe |
| India | 021 | | Life Care Institute of medical sciences | Sameer Dani |
| India | 022 | | CIMS Hospital, Ahmedabad | Keyur Parikh |
| India | 023 | | Sanjivani Hospital, Ahmedabad | Kamal Sharma |
| India | 024 | | Baroda Heart Institute | Parvinder Singh |
| India | 025 | | Heart First Cardiac and Vascular Centre | Atul Abhyankar |
| India | 026 | | Star Hospital, Hyderabad | Ramesh Gudapati |
| India | 027 | | Prime Hospital, Hyderabad | C Raghu |
| India | 028 | | Apollo Hospitals, Bilaspur | M P Samal |
| India | 029 | | K M C, Manipal | Tom Devasia |
| India | 030 | | Jayadeva Institute of Cardiology, Bangalore | Dr. Prabhavathi |
| India | 031 | | Narayana Hrudayalaya, Bangalore | Srikanth K.V. |
| India | 032 | | Madras Medical Mission | Ajit Mullasari |
| India | 033 | | Kakadiya Hospital, Ahmedabad | Bhavesh Thakkar |
| India | 034 | | International Hospital, Guwahati | Neil Bordoloi |
| India | 035 | | Frontier Lifeline Hospital, Chennai | Nagendra Prasad |
| India | 036 | | Meenakshi Mission Hospital, Madurai | R Sivakumar |
| India | 037 | | Isabel Hospital | K.P.Shamsuddeen |
| India | 038 | | Niramaya Hospital, Pune | Abhijeet Joshi |
| India | 039 | | PRS Hospital, Trivandrum | Tiny Nair |
| India | 040 | | Kerala Institute of Medical Sciences, Trivandrum | Govindan Vijayaraghavan |
| India | 041 | | Lakeshore Hospital, Cochin | Anand Kumar |
| India | 042 | | K G M C, Lucknow | V S Narain |
| India | 043 | | S G P G I, Lucknow | Aditya Kapoor |
| India | 044 | | Pargaonkar Hospital & Heart Care Centre | Kishore Pargaonkar |
| India | 046 | | Mittal Hospital & Research Center, Ajmer | Rahul Gupta |
| India | 047 | | G B Pant Hospital, New Delhi | Vimal Mehta |
| India | 048 | | Batra Hospital and Medical Research Centre, New Delhi | Rajeev Bajaj |
| India | 049 | | Chirayu Cardiac Centre, Bhopal | R K Singh |
| India | 050 | | Crescent Hospital & Heart Centre | Aziz Khan |
| Malaysia | 001 | | Sarawak General Hospital Heart Centre | Ong Tiong Kiam |
| Malaysia | 002 | | Penang Hospital | Leong Weng San |
| Singapore | 001 | | National Heart Centre Singapore | Chin Chee Tang |
| South Korea | 001 | | Seoul National University Hospital | Hyo-Soo Kim |
| South Korea | 002 | | Boramae Medical Center | Sang-Hyun Kim |
| South Korea | 003 | | Gangnam severance | Kwon Hyuck Moon |
| South Korea | 004 | | Dong-a University Medical Center | Young-Dae Kim |
| South Korea | 005 | | Pusan National University Yangsan Hospital | Kook Jin Chun |
| South Korea | 006 | | The Catholic University of Korea, Seoul St. Mary's Hospital | Pum Joon Kim |
| South Korea | 007 | | Kangbuk Samsung Hospital | Jin Ho Kang |
| South Korea | 008 | | Gwangju Veterans Hospital | Won Yu Kang |
| South Korea | 009 | | Kosin University Gospel Hospital | Tae Joon Cha |
| South Korea | 010 | | Wonkwang University Hospital | Seok Kyu Oh |
| South Korea | 011 | | Jeju National University Hospital | Seung Jae Joo |
| South Korea | 012 | | Chuncheon Sacred Heart Hospital | Hyun-Hee Choi |
| South Korea | 013 | | Deagu Catholic University Medical Center | Jae Kyun Ryu |
| South Korea | 014 | | Chung-Ang University Hospital | Sang Wook Kim |
| South Korea | 015 | | Wallace Memorial Baptist Hospital | Joon-Sang Lee |
| South Korea | 016 | | Sejong Hospital | Young-Jin Choi |
| South Korea | 017 | | Inje University Haeundae Paik Hospital | Doo-il Kim |
| South Korea | 018 | | The Catholic University of Korea, St. Vincent's Hospital | Keon-Woong Moon |
| South Korea | 019 | | Dankook University Hospital | Myoung Yong Lee |
| South Korea | 020 | | Inje University Ilsan Paik Hospital | Sung Yun Lee |
| South Korea | 021 | | Kangdong Sacred Heart Hospital | Kyoo-Rok Han |
| South Korea | 022 | | The Catholic University of Korea, Yeouido ST. Mary's Hospital | Chul Su Park |
| South Korea | 023 | | Severance Hospital | Byeong-Keuk Kim |
| Thailand | 101 | | Siriraj Hospital | Rungroj Krittayapong |
| Thailand | 102 | | King Chulalongkorn Memorial Hospital | Suphot Srimahachota |
| Thailand | 103 | | Ramathibodi Hospital | Piyamitr Sritara |
| Thailand | 104 | | Thammasat University Hospital | Dilok Piyayothai |
| Thailand | 105 | | Phramongkutklao Hospital | Chumpol Piamsomboon |
| Thailand | 106 | | Police General Hospital | Kasem Ratanasumawong |
| Thailand | 107 | | Bhumibol Adulyadej Hospital | Thanavit Sakulseangprapa |
| Thailand | 108 | | Piyavate Hospital | Nithi Mahanonda |
| Thailand | 109 | | Phyathai 2 Hospital | Odthon Sriyudthasak |
| Thailand | 110 | | Maharaj Nakorn Chiangmai Hospital | Srun Kuanprasert |
| Thailand | 111 | | Chiangmai Ram Hospital | Pattarapong Keelapan |
| Thailand | 112 | | Srinagarind Hospital | Chaiyasith Wongwipaporn |
| Thailand | 113 | | Maharaj Nakornratchasima Hospital | Pinij Keawsuwanna |
| Thailand | 114 | | Chonburi Hospital | Pornchai Ngamjanyaporn |
| Thailand | 115 | | Buddhachinaraj Hospital | Poj Jianmongkol |
| Thailand | 116 | | Prapokklao Hospital | Thanwa Pituksuthipong |
| Thailand | 117 | | Songklanagarind Hospital | Noppadol Chamnarnphol |
| Thailand | 118 | | Vachira Phuket Hospital | Nara Kingkeaw |
| Thailand | 119 | | Suratthani Hospital | Suthep Terdudomtham |
| Thailand | 120 | | Chaophrayayomraj Hospital | Pairoj Pinjeesekikul |
| Thailand | 121 | | Chiangraiprachanukroh Hospital | Watthana Wongtheptien |
| Thailand | 122 | | Lampang Hospital | Thanita Boonyapiphat |
| Thailand | 123 | | Nan Hospital | Niwatchai Sucharitchan |
| Thailand | 124 | | Udonthani Hospital | Sumon Tangsuntornwiwat |
| Thailand | 125 | | Khonkean Hospital | Sudhithep Duangsorn |
| Vietnam | 001 | | Cho Ray Hospital | Vo Thanh Nhan |
| Vietnam | 002 | | NDGD hospital | Hoang Quoc Hoa |
| Vietnam | 003 | | 115 Hospital | Pham Duc Dat |
| Vietnam | 004 | | HCMC University Medical Centre | Truong Quang Binh |
| Vietnam | 005 | | Tam Duc Heart Hospital | Dinh Duc Huy |
| Vietnam | 006 | | HCMC Heart Institute | Do Quang Huan |
| Vietnam | 007 | | Trung Vuong Hospital | Do Quang Huan |
| Vietnam | 008 | | Hue Cardiovascular centre | Nguyen Cuu Loi |

Supplementary Table SII. Mean (95% CI) individual center-specific cost ($INT) per procedure by country*

| **Region/Country** | | | | | | | |
| --- | --- | --- | --- | --- | --- | --- | --- |
|  | **China**  **(*n* = 7704)** | **Hong Kong**  **(*n* = 125)** | **India**  **(*n* = 2283)** | **Singapore**  **(*n* = 65)** | **South Korea**  **(*n* = 247)** | **Thailand**  **(*n* = 235)** | **Vietnam**  **(*n* = 160)** |
| ECG | 7.6  (7.1, 8.0) | 58.9  (51.4, 66.5) | 9.9  (8.7, 11.2) | 130.5  (NA) | 9.9  (7.2, 12.6) | 22.8  (17.1, 28.5) | 3.3  (1.9, 4.7) |
| Cardiac markers | 59.5  (54.6, 64.2) | 69.5  (56.4, 82.4) | 82.9  (71.4, 94.4) | 313.2  (NA) | 39.5  (29.4, 49.5) | 40.1  (30.8, 49.2) | 21.7  (2.5, 40.5) |
| Echocardiography | 97.5  (76.9, 118.1) | 352.8  (306.0, 399.6) | 78.2  (68.6, 87.9) | 1651.6  (NA) | 240.9  (218.3, 263.6) | 187.4  (170.0, 204.4) | 25.8  (15.8, 35.8) |
| Angiography | 1205.3  (1108.9, 1301.5) | 2071.5  (1101.7, 3041.3) | 1067.4  (632.1, 1502.7) | 17425.8  (NA) | 869.2  (490.0, 1248.4) | 1669.6  (1395.6, 1943.7) | 788.9  (582.4, 995.4) |
| PCI with 1 drug eluting stent | 5557.8  (5101.6, 6014.1) | 5878.9  (3526.8, 8231.1) | 11477.3  (6990.9, 15964.0) | 65579.7  (NA) | 2581.3  (1908.4, 3254.4) | 11027.6  (8945.4, 13109.9) | 8974.9  (7575.9, 7598.4) |
| PCI with 1 bare metal stent | 3933.8  (3481.6, 4386.1) | 2619.6  (832.4, 5011.0) | 6806.2  (5770.2, 7842.3) | 29807.2  (NA) | 2283.5  (1682.8, 2884.3) | 8404.1  (6751.5, 10056.4) | 5274.0  (4281.9, 6266.0) |
| CABG | 297.5  (216.5, 378.4) | 10197.0  (5891.1, 14502.9) | 10137.3  (9060.9, 11213.4) | 110057.5  (NA) | 5810.0  (3752.0, 7868.1) | 18737.3  (11608.0, 25866.6) | 11547.2  (8272.1, 14822.4) |
| Stay in CCU per day | 297.5  (216.5, 378.4) | 1998.7  (1663.8, 2333.5) | 281.6  (225.1, 337.8) | 3210.0  (NA) | 160.5  (95.6, 225.4) | 283.9  (153.5, 414.0) | 98.0  (12.2, 200.7) |

*CABG* coronary artery bypass graft, *CCU* critical care unit, CI, confidence interval; *ECG* electrocardiogram, *NA* not available, *PCI* percutaneous coronary intervention.

*Exchange rate conversion based on (March 2013): 1 USD = 6.2943 CNY; 7.7681 HKD; 53.060 INR; 3.1690 SGD; 1158.1 KRW; 31.545 THB; 21030 VND. The $US figures converted into $INT using the following purchasing power parity conversion factors (2015): 3.48 CNY, 5.76 HKD, 17.09 INR, 0.85 SGD, 888.73 KRW, 12.19 THB, and 7576.25 VND.
